# Surrogate consent for critical care research: exploratory study on public perception and influences on recruitment

**DOI:** 10.1186/cc11927

**Published:** 2013-01-15

**Authors:** Daphne AFN Lim, Moon Fai Chan, Charmaine Childs

**Affiliations:** 1 Alice Lee Centre for Nursing Studies, Yong Loo Lin School of Medicine, National University of Singapore and National Health System, Level 2, Clinical Research Centre, Block MD11, 10 Medical Drive, 117597, Singapore

## Abstract

**Introduction:**

Critical Care research involves an increasing level of technical and clinical interventions for the unconscious patient. If the general public has a negative (unfavourable) view of surrogate consent, low recruitment rates are likely. Results bias will be introduced if study populations are small, hindering knowledge generation and transfer through research. In the rapidly expanding healthcare industry of South East Asia, opportunities for critical care research will grow given a positive willingness (favourability) by the general public to act as a surrogate in the consent process when the (unconscious) patient cannot consent for him/herself.

**Methods:**

To determine public willingness for surrogate consent, a quantitative cross-sectional study was undertaken at a University Teaching Hospital in South East Asia during a three month interval. Four hypothetical critical care research scenarios were presented and responses from the public were analysed using a three-part questionnaire.

**Results:**

Three hundred and five members of the public were recruited. In general, participants had a positive view of research. The level of education was significantly associated with a person's views about research especially in studies regarded as high risk. For low risk studies, a person's perception of research and willingness to be recruited to a study in the event that they were the (unconscious) patient, was the same whether they were the study subject or the person (legally acceptable representative) giving surrogate consent' on behalf of another (spouse, parent, child). Across all study scenarios, 60-80% of the public preferred to be approached by doctors to discuss the surrogate consent process.

**Conclusion:**

Given the hypothetical scenarios presented in this study, the odds of a person having a positive view and willingness to be consented to a critical care research study on the advice of another (surrogate consent) was greater than for those who had a negative or unfavourable view. Nurses may be disadvantaged in leading on the recruitment process due to a preference for information to be delivered by medically qualified clinicians. In the setting of South East Asia, cultural attitudes to nurse-led research in critical care must be taken in to consideration in the multidisciplinary approaches to building the research team.

## Introduction

Recruitment of critically ill patients is essential to undertake critical care research. However, the patient frequently lacks capacity to decide for him/herself whether to give informed consent for participation in clinical research. To counteract the barrier that would otherwise exclude research from being undertaken on unconscious patients, legislation in many countries allows the patient's relative or other appropriate person to give surrogate consent [1]. There are several strategies whereby ethical approval is obtained: it may be deferred until consciousness is regained, waived entirely, obtained in advance (if a directive is available) or it may be obtained from a surrogate acting on behalf of the unconscious patient [2].

Whilst many people support clinical research, those who do not have a positive view of human research may present a barrier to recruitment. As a fundamental principal, participation requires the patient's representative to provide a witnessed signature to consent as a surrogate or legally acceptable representative (LAR) of the patient [3]. Most commonly, the reason for a positive view towards research includes altruism and a belief of a way forward for medical science [3]. By contrast, the often quoted negative view that research allows people to be treated like 'guinea pigs' [3,4] remains within contemporary society and has a negative impact for fertile discussion. Preliminary findings obtained during the course of an observational low-risk study in a surgical ICU produced a 50% recruitment rate only (personal communication). This contrasts with experiences of a successful recruitment rate in a perceived higher-risk interventional study [5] in an intensive care setting in Europe. Of concern for the future of critical care research recruitment in South East Asia (especially with the burgeoning interest in studying the Asian phenotype across different clinical and healthcare settings) is the ability to achieve pre-defined target recruitment rates. The aim of the study therefore, was to determine perceptions towards surrogate consent in a sample population of the general public.

The objectives were to: 1) determine public opinion and views about healthcare research in a sample of the local population; 2) test whether public perception towards surrogate consent differs with respect to the perceived level of risk of the study, age, gender and level of education 3) determine parity between an individual's perception towards surrogate consent (that is, a person's own views on a relative giving consent on their behalf should they be unconscious in an ICU) versus their perception of giving consent on behalf of another (that is, being the LAR for an unconscious relative admitted to intensive care), and 4) determine the perception of the public with regard to the most appropriate health worker to approach an LAR to obtain surrogate consent in critical care research.

## Materials and methods

### Study design and participant sample

This was a quantitative, exploratory, cross-sectional study with convenience sampling, undertaken during November 2011 to January 2012. Members of the general public in the environs of the University Hospital, aged ≥ 21 years and conversant in English (and able to read and write English) were eligible for study recruitment.

### Setting and Questionnaire

Members of the public attending a large university teaching hospital out-patient clinics, or visitor public waiting areas were invited to participate in the study after a brief introduction by the researcher about her role in the study and the purpose of the information requested.

A three-part questionnaire was used for data collection. The questionnaire was adapted from a published study exploring surrogate consent in patients suffering from dementia [6]. For each participant, sociodemographic information was obtained followed by responses to a nine-item Research Attitudes Questionnaire (RAQ). A five-point (1 to 5) Likert scale was used to measure general favourability towards healthcare research [6]. The nine-item RAQ gives a possible score range of 9 to 45.

To obtain information about public willingness towards surrogate consent in the setting of critical care, all participants received a standardised verbal explanation guided by a script. The participant was informed that a personal view and reflection, favourable (positive) or unfavourable (negative), was required under both the following two broad circumstances, specifically relating to a scenario whereby:

1. You are visiting a relative receiving treatment in the ICU. During the visit you are approached and asked to give permission for your relative to take part in a research study being undertaken on the ICU where your relative is being treated.

2. You are (this time the unconscious patient) admitted to the ICU. Your relative is asked to consider whether in their opinion, you (as the patient) would be willing and would agree to participate in a research study even though you are unconscious and therefore unable to speak for yourself.

To illustrate the type and nature of research being proposed, four hypothetical studies were described verbally. The scenarios were also written in a questionnaire. In each of the four scenarios, questions (items) were listed. The research study scenarios were categorised as a minimally invasive study, a non-invasive observational study, an invasive observational study and a drug trial (Table 1). Participants were asked to rate their responses to each of the scenarios as negative (unfavourable) meaning they would definitely not allow or would probably not allow consent, or positive (favourable) meaning they would probably allow or would definitely allow consent. For statistical purposes the two favourable options (probably or definitely allow consent) were combined to represent positive perceptions, while unfavourable options (definitely not or probably not allow consent) were combined to represent negative perceptions, for the purposes of analysis. In addition, three of the seven questions allowed for an open-ended response from participants to elaborate their opinion and attitudes, to give a qualitative evaluation of responses. After the researcher had explained the study, the participant was guided through the written study items and scenarios. Consent was then obtained. Thereafter, the participant was given the opportunity to complete the questionnaire in their own time, whereupon it was handed back to the researcher.

**Table 1:** Research study category, scenarios used and brief descriptor

Research study category	Name of research scenario	Short description
Minimally invasive study (low risk)	Blood draw study	Drawing of a small amount of blood for genetic research from an existing intravenous cannula
Non-invasive observation study (low risk)	Mattress study	Testing of a new pressure-relieving mattress
Invasive observational study (high risk)	Brain sensor study	Insertion of additional brain sensors for brain tissue monitoring in brain-injured patients in addition to routinely used brain sensors
Drug trial (high risk)	Drug trial	Double-blind drug randomized controlled trial where the drug in the scenario was explained to possibly cure cancer in cancer patients

### Statistics and study power analysis

The target participant sample was calculated based on the odds ratio (OR) for greater willingness (favourability) to allow surrogate consent for a blood draw (perceived low-risk) and drug trial (perceived high-risk) scenario respectively [6]. The adjusted ORs were 3.61 and 2.70 respectively. To achieve 80% power at the 5% significance level, the desired participant sample was between 167 and 279 participants. The chi-square and Student's *t*-test was used to determine differences between groups as appropriate. Reverse-worded items in the RAQ were reverse-scored using the transform and recode functions in Statistical Package for the Social Sciences (SPSS) software. Scores were recorded as mean and SD.

### Ethics approval

Local research ethics approval was obtained before commencement of the study. Approval was obtained from senior management to conduct the study on hospital premises. Data were anonymous. No personal identifiers were recorded.

## Results

### Participants

Of 305 participants, an equal distribution of male to female individuals were recruited (151 (49.5%) male, 154 (50.5%) female), with ages ranging from 21 to 81 (median 42) years. The response rate was 74.2% (305/411 questionnaires distributed). The majority of participants were Chinese (*n *= 199, 65.2%) and most were married (*n *= 200, 65.6%). An equal number of participants had a higher education diploma (*n *= 87, 28.5%) or Bachelor's degree (*n *= 87, 28.5%). Two-thirds (*n *= 104, 34.1%) were in managerial or technical and professional occupations (*n *= 96, 31.5%) (Table 2).

**Table 2:** Sample characteristics of participants (total recruited = 305)

Sample Characteristics	Number	%
**Age range: 21 to 81 (median 42) years**	305	100.0
**Gender**		
Male	151	49.5
Female	154	50.5
**Ethnic group**		
Chinese	199	65.2
Malay	36	11.8
Indian	40	13.1
Eurasian	11	3.6
Others*	19	6.2
**Marital status**		
Single	99	32.5
Married	200	65.6
Divorced	6	2.0
**Highest level of education**		
Primary school	7	2.3
Secondary school	64	21
Junior college	26	8.5
Diploma	87	28.5
Bachelors degree	87	28.5
Masters degree	24	7.9
Doctoral degree (PhD, MD)	10	3.3
**Occupation^‡^**		
I) Professional	96	31.5
II) Managerial/technical	104	34.1
III) Unskilled	10	3.3
IV) Student	25	8.2
V) Unemployed and homemakers	22	7.2
VI) Retired	22	7.2
VII) Others (withheld, self-employed et cetera)	26	8.5

^‡^Registrar General Scale of Social Class [17]. *Includes Indonesian, Caucasian, Arab.

### General attitudes towards clinical research

Individual RAQ scores for items 1 to 9 are given in Table 3. For each item, average values were obtained. The three highest scores for the participants' perception (favourability) of healthcare research were for a positive societal perception that is, recognition that society needs to devote more resources to medical research (item 5), a positive personal view about medical research (item 1) and the belief that medical research will find cures for current prevalent diseases in the future (item 9, Table 3). For the RAQ scoring system, reverse-worded items (items 2, 4, 6 and 8) were reverse-coded to calculate the overall mean score. Of a possible maximum score of 45, the mean (SD) score was 29.28 (3.59).

**Table 3:** Research Attitudes Questionnaire: mean scores

Items	Mean score	SD
1. I have a positive view about medical research in general.	3.93	0.74
2. Medical researchers are mainly motivated by personal gain.^†^	3.08	0.94
3. Medical researchers can be trusted to protect the interests of people who take part in their research studies.	3.46	0.80
4. Modern science does more harm than good.^†^	3.37	0.91
5. Our society needs to devote more resources to medical research.	3.95	0.75
6. The government needs to closely regulate medical research in order to prevent harm to research participants.^†^	1.65	0.75
7. Medical research involving humans is by and large safe.	3.23	0.79
8. Putting too much emphasis on medical research and scientific progress is likely to harm research volunteers who cannot look after their own interests.^†^	2.93	0.90
9. Medical research will find cures for many major diseases during my lifetime.	3.68	0.81
Total (of 45*)	29.28	3.59

^†^Shows reverse-worded items; each reverse-worded item was reverse-coded to calculate the overall mean score. *The total score does not correspond with a cutoff point definition; the total score merely reflects the overall score obtained from the individual's responses for each item likert scale.

### How does an individual's perception of study risk influence their own attitude to research recruitment in the event that they are the unconscious patient?

For the hypothetical blood draw and mattress study scenarios, participants were five times (OR 5.19, 95% CI 2.72, 9.92) and six times (OR 6.36, 95% CI 3.22, 12.56) more likely to have a positive (favourable) perception about themselves being the unconscious research participant for the blood draw (*P *< 0.001) and mattress (*P *< 0.001) studies respectively, when they perceived the study as low risk compared to those who perceived these types of study as high risk (Table 4). That is, the participants interviewed had a positive attitude and willingness for a family member to act as an LAR to give surrogate consent for research on their behalf, in the event that they themselves were unconscious in the ICU.

**Table 4:** Participants' perceived willingness to be recruited by an LAR to critical care studies at different levels of risk

		Participants' views on study risk					
						
Study scenario	Participant-perceived risk	Positive Number (%)	Negative Number (%)	Statistic^	*P*-value	OR	95% CI
Blood draw	Low risk	206 (80.2)	21 (43.8)	28.16	< 0.001	5.19	2.72, 9.92
	High risk*	51 (19.8)	27(56.2)				
Mattress	Low risk	186 (72.4)	14 (29.2)	33.45	< 0.001	6.36	3.22, 12.56
	High risk*	71 (27.6)	34 (70.8)				
Brain sensor insertion	High risk	128 (71.1)	117 (93.6)	23.61	< 0.001	5.94	2.71, 13.0
	Low risk*	52 (28.9)	8 (6.4)				
Drug trial	High risk	134 (70.2)	101 (88.6)	13.73	< 0.001	3.31	1.72, 6.37
	Low risk*	57 (29.8)	13 (11.4)				

^Chi-square test; *reference category for odds ratio (OR).

In the brain sensor and drug trial scenarios, 70% of participants who perceived these studies as high risk were six times (OR 5.94, 95% CI 2.71, 13.0) and three times (OR 3.31, 95% CI 1.72, 6.37) more likely to have a positive perception (favourability) towards being recruited to such a study (brain sensor, *P *< 0.001 and drug trial, *P *< 0.001 respectively) on the opinion of an LAR surrogate, compared to those who perceived the hypothetical studies as low risk (Table 4). Positive attitudes to the hypothetical study scenarios were evident across all levels of perceived study risk. Where study scenarios were categorised as higher risk, the favourability of the participant to be willing to be a research subject if they themselves were an unconscious patient in the ICU (71% and 70% for the brain sensor and drug trials respectively (Table 4) is explained to some extent by the attitudes towards research of the subjects themselves (Table 5)

**Table 5:** Narratives from participants obtained from open-ended questions about consenting to high-risk studies

Study ID	Brain sensor study	Study ID	Drug Trial
146	Scenario already so traumatic, trying out this trial will not make a difference	41	If I am terminally ill and have exhausted all therapy, yes
188	While this study may impose some risks on me, I am also not guaranteed of consciousness or cure, With that, I'd rather decide for the greater good of people with higher chances of surviving than I do	100	Nothing to lose, patient is already unconscious
216	I believe the doctors will make a recommendation that is beneficial to me and my family will act based on the doctor's recommendation	146	Patient is already critically ill, worth to try out the new drug
234	This study gives doctors better observation on my brain	150	My family would like to try it out to see if the drug can cure me
236	It helps to have additional observational data and can detect deterioration	154	No harm as I am unconscious in the scenario
243	This study can help knowledge of my illness	168	Give the new drug a shot to cure me
291	Patient is already unconscious. It is OK to test on me. However, I think my family member will probably say 'NO' if it was my children deciding for me	184	It is a good idea to test new drugs to know its effectiveness
		217	I am more interested in how the drug can cure me
		234	Will only try if there are no other treatment choices
		236	I am willing to try, better than doing nothing about my condition
		238	If you don't try, you don't know the effects of the drug
		243	To take my chance for a cure

The narratives explain why participants' views about themselves participating in critical care research were favourable (positive) when the study was categorised as high risk.

### What factors affect a person's perception of surrogate consent?

The hypothetical scenario that we have framed as: 'Suppose you become critically ill in the future and cannot make a decision for yourself about whether you wish to take part in a critical care study," reveals that level of education was the only factor that significantly influenced a person's view about whether they would be willing to participate in a critical care study if they were the unconscious patient. There were no significant associations between a person's own view about surrogate consent and age, gender or marital status. Whilst the level of education was shown to have an association with a person's willingness towards surrogate consent, this was significant only for the more invasive research scenarios (brain sensor and drug trials). When responses were compared between those with positive versus negative views (perceptions) about participating in the brain sensor study, the odds for participation doubled for those with secondary school (or lower) education (*P *= 0.031, 95% CI 1.06, 3.73 compared with degree education (OR 1.99 for secondary versus degree education). Similarly, for the drug trial scenario, positive views (willingness) to participate in the drug trial doubled for those with secondary school (or lower) education compared to degree education (OR 1.94, 95% CI 1.01, 3.70, *P *= 0.044). Less educated people (secondary school or lower) had a more favourable approach to participating in the high-risk brain sensor and drug trial studies.

### Is a person's perception of surrogate research the same for him/herself as it is for other close family members?

When participants had a positive and favourable perception about being recruited to a critical care research study they had similar positive views about giving consent as an LAR for their spouse, parent and child being enrolled in an ICU study, but this was for the blood draw and mattress studies only (Table 6).

**Table 6:** Participants' perceptions' of their own recruitment to studies with different levels of risk compared with their willingness to give surrogate consent for recruitment of a family member to similar studies

		Participants' own views on participation for their spouse, parent or child											
		
		Spouse				Parent				Child			
		
Scenario	Perception (favourability) for him/her self to be recruited by an LAR	Yes N (%)	No N (%)	OR	95% CI	Yes N (%)	No N (%)	OR	95% CI	Yes N (%)	No N (%)	OR	95% CI
Blood draw study	Positive	186 (86.1)	12 (60.0)	4.13	1.56, 10.95	169 (82.0)	26 (81.3)	1.05	0.41, 2.74	133 (86.9)	12 (57.1)	4.99	1.87, 13.34
	Negative*	30 (13.9)	8 (40.0)			37 (18.0)	6 (18.7)			20 (13.1)	9 (42.9)		
Mattress study	Positive	193 (87.7)	11 (64.7)	3.90	1.33, 11.40	181 (86.2)	21 (70.0)	2.68	1.12, 6.41	149 (92.5)	8 (57.1)	9.31	2.77, 31.26
	Negative*	27 (12.3)	6 (35.3)			29 (13.8)	9 (30.0)			12 (7.5)	6 (42.9)		
Brain sensor study	Positive	127 (62.0)	16 (51.6)	1.53	0.72, 3.26	111 (58.1)	29 (58.0)	1.01	0.54, 1.89	95 (66.0)	17 (60.7)	1.26	0.55, 2.89
	Negative*	78 (38.0)	15 (48.4)			80 (41.9)	21 (42.0)			49 (34.0)	11 (39.3)		
Drug trial	Positive	143 (67.5)	12 (48)	2.25	0.97, 5.18	119 (59.8)	24 (58.5)	1.05	0.53, 2.09	99 (68.8)	15 (65.2)	1.17	0.46, 2.97
	Negative*	69 (32.5)	13 (52.0)			80 (40.2)	17 (41.5)			45 (31.2)	8 (34.8)		

LAR, legally acceptable representative; OR, odds ratio; *reference category for odds ratio; N, number.

### Who do people believe to be the preferred healthcare worker to discuss the research consent process?

**Figure 1:**
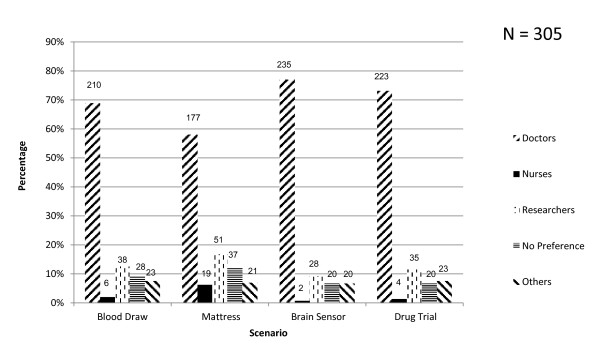
**Surrogate choice for preferred healthcare worker to discuss consent for research**. Numbers of respondents are shown above the histogram bars. Others are participants who selected more than one option.

For all scenarios the participant's preference for the most appropriate person to talk to them with regard to seeking consent for a loved one to enter a critical care research study was a doctor: *n *= 210 (68.9%) for the blood draw study, *n *= 177 (58%) for the mattress study, *n *= 235 (77%) for the brain sensor study, and *n *= 223 (73.1%) for the drug trial. Few participants considered nurses or researchers to be the preferred person to talk to them about surrogate consent for research. For 'others', the choice was always a combination of a doctor plus another healthcare professor; nurse or researcher (Figure 1).

## Discussion

The concept of surrogate consent originated with the Belmont Report [7], the objective being to protect the interests of unconscious patients who lack the capacity to decide any matter for themselves, including their participation in healthcare research. The decision, therefore, becomes the responsibility of another person. This person then adopts a status as an LAR, and due to legislation in many countries worldwide, the LAR is allowed to give consent for a close family member or friend to be recruited to a clinical trial. There are reasons and circumstances governing an individual's decision to give consent on behalf of another (in this case consent for research) but above all, it is determined by the person's own perception of the request and their willingness to condone it. In this regard, the nature of the research being conducted is important [3] in determining a person's perception (positive or negative) of being comfortable to give surrogate consent. One common reason for negative feelings is the view that research allows people to be treated as 'guinea pigs' [3,4].

In the population of participants recruited to the current study there was a positive view about healthcare/medical research. The RAQ scores favoured attitudes that embrace research, exemplified by the highest scores for; 'our society needs to devote more resources to medical research' and 'I have a positive view about medical research in general'. As Kim *et al. *state, 'People's general attitude towards research is a strong predictor of acceptability of surrogate consent for research." [6]. How then does this general attitude to research influence willingness to give consent on behalf of another? Research evidence suggests that surrogates struggle as decision-makers for the unconscious patient [8]. This results in less confidence in functioning as a surrogate [8], which could possibly lead to a negative attitude towards research and willingness to give consent on behalf of another family member.

By contrast, one of the common reasons for a positive attitude and willingness to give surrogate consent is altruism [3,4]. Should the relative have negative connotations of the notion of medical research, he/she may not be able to appreciate the altruistic reasons underpinning study recruitment. Of importance in the context of this current study, however, is a potential mismatch between the hypothetical scenario and real-world conditions. Here the distress and anxiety of sudden, unexpected illness in a relative or close friend may lead to a change in attitude; positive views may become negative when the surrogate is under stress [9]. It is at this time that the level of risk of the study under discussion is likely to be of importance in influencing decisions about surrogate consent [10]. In support of this view we have shown that participants who perceive a study as low risk were more likely to have a positive perception towards surrogate consent. It is also possible that familiarity with a procedure may also influence perception of risk [11]. For example, even though the blood draw scenario we have illustrated is essentially an invasive procedure, it yielded the highest proportion of people who thought taking blood was low risk. The participants also had a concomitant positive self-perception towards surrogacy compared to the other scenarios.

When we provided a scenario that we consider high risk (brain sensor and drug trials), of those participants who also perceived these interventions as high risk, many had positive perceptions towards surrogate consent. High-risk scenarios were defined as studies anticipating a 'magnitude of harm greater than ordinarily encountered in daily life or during performance of routine physical examinations' [12]. The same view was shown previously to apply to research scenarios that involved testing of new equipment or a drug [3,10]. Here, uncertainties in terms of benefit were outweighed by the harm that might be brought to patients, linking higher risk with negative perceptions towards surrogate consent. In this study, review of the open-ended questions clearly indicates that despite the potential risks involved, many people perceive the risks to be acceptable.

Responses obtained from participants to open-ended questions gave us insight as to why participants still have a positive perception towards surrogate consent despite the potential risks of the research studies. For example, in the brain sensor study, an individual stated that (participating in the study) '... helps to have additional observational data and can detect deterioration.' Whereas, in the drug trial scenario, an individual stated that he was '... willing to try, (which is) better than doing nothing about my condition.' (Table 5) Whilst there is a perception from people who are approached to act as a surrogate on behalf of another, we did not find any comment supporting the 'guinea pig' [4] viewpoint about participation in research that was likely to affect the relative's decision-making process on behalf of another [6].

For the current study, it was the brain sensor scenario where participants' perceptions of high versus low risk was greatest, with participants six times more likely to perceive the study as high compared to low risk. This view may be influenced by the organ (brain) under investigation. Here cultural attitudes to interfering with the brain may be shrouded in suspicion and mystery, leading to an aversion to interference of any kind. Furthermore, whilst personal feelings of altruism on the one hand and the harbouring of negative feelings of suspicion and mistrust on the other all contribute to a person's perception of research and willingness to participate, other factors can also influence decision-making. We have shown that of the sociodemographic factors investigated, educational level was significantly associated with a person's perception towards surrogate consent in the higher risk studies (brain sensor and drug trial scenarios). Here, participants with an education up to secondary school level were more likely to have a positive perception towards surrogate consent than those who held a degree-level qualification. With a higher level of education, people may reflect and weigh up the cost and benefit of research studies, especially when it may be perceived that there is a degree of risk. In a society where education is greatly valued, people are able to think independently, have their own views and opinions about research and become more questioning when approached for consent for research participation on behalf of a relative or spouse. Participants who reported a positive perception towards surrogate consent were more likely to have the same perception for their family member, supporting the work of Kim *et al. *[6] but with the caveat that this was for lower risk studies only.

In this study population, the majority of participants preferred to have a doctor approach them when consent is being sought on behalf of an unconscious family member. This finding was consistent across all the study scenarios and tends to show some support of the view about the lower status nurses hold compared to doctors in Asia [13]. People in the East generally accept the paternalistic role doctors play in the healthcare field [14]. The majority of participants may have preferred a doctor to approach them when seeking consent on behalf of another because of the perception that advice and information from doctors is more reliable or more accurate. This illustrates the potential difficulties and limitation in scope that nurse researchers and academics face in leading clinical research. Most notably perhaps, and due to the complexity of the patient's condition and treatment regimen, nurse-led critical care research may reach a roadblock without the participation of a clinician to actively participate in the discussions of surrogate consent.

### Limitations

As the questionnaire was produced in English, limited conversational fluency in Mandarin or other dialects prevented the study from being undertaken by members of the public who were not conversant in English. In this regard, the results do not give an accurate representation of the general public's perception towards surrogate consent [15]. It is therefore recommended that a larger study be undertaken for a fuller representation of the topic in the context of South East Asia.

Because of the specific focus of the study, we reflected on the need to directly approach the relatives of critical care inpatients to elicit their views on surrogate consent for critical care research, but in the local setting our experience is that this would be intrusive and not well-received during what is well-recognised as a stressful and distressing period for relatives of ICU patients. As an alternative first step, members of the public within the grounds of the hospital were sought. The limitation of this approach was that participants were not in the real-state distress experienced by so many family members of an unconscious patient. Whilst the scenarios presented are examples of genuine studies, for the participants they were in effect hypothetical [16]. Thus the responses may give rise to different perceptions as would occur in the event of a real-life situation, either for the participant as a patient in the ICU being recruited to a study on the consent of another, or as the participant per se acting as the LAR on behalf of a family member.

To overcome the limitations of bias in the sample population, even with the large sample population recruited, further investigation would require a greater breadth of the population. We also believe that the demographics of the study population we have obtained may over-represent professional and managerial occupations. A future study would need to reach the full gamut of occupational and social classes of the local population, supported by qualitative data and in-depth thematic analysis of participant narratives where appropriate.

### Implications for practice

In the context of nurse-led research in the setting of critical care, the findings from this study, suggest that nurses may be at a disadvantage from the perspective of autonomous research leadership. In the short-term, research teams will require a physician to be an active member of the team. For the future, an increase in the status of nursing should provide a greater opportunity for nurses to fulfil their role and to contribute to the full breadth of clinical science underpinning evidence-based practice.

We recommend creating awareness among the public about research participation by unconscious patients in critical care, emphasising the role and importance of the LAR. With greater awareness, relatives may act based on their actual views towards research participation for unconscious patients, and not regard the act of being approached for surrogate consent as additional stress and trauma to that which family members experience when a loved one is critically ill.

## Conclusions

People with positive views about clinical research are more likely to agree to be a participant in critical care research, and more likely to give their consent (as a surrogate) for recruitment of a family member especially if the study is low risk. Nurses may be disadvantaged in leading on the recruitment process due to a preference for information to be delivered by medically qualified clinicians. In the setting of South East Asia, cultural attitudes to nurse-led research in critical care must be taken in to consideration when building multidisciplinary research teams.

## Key messages

• People who have a positive and favourable view about research are more willing to give their consent to a critical care research study compared to those with negative or unfavourable views of research.

• Healthcare professionals should be aware that people with a basic education may have a more favourable view about research. The reasons are unclear. Researchers need to be mindful to give the LAR a complete and understandable account of the study to avoid unintended coercion.

• People who are willing to be recruited to a research study in the setting of critical care are also likely to agree to their spouse, parent or child participating in the same type of study, but only if it is viewed as low risk.

• With the research scenarios illustrated in the current study, doctors were preferred as the member of the healthcare team most appropriate to discuss issues of surrogate consent for research. The public perception of nurses to lead on the consent process was poor. This has implications for allied health professionals to operate as independent clinical researchers.

## Abbreviations

LAR: legally acceptable representative; RAQ: Research Attitudes Questionnaire.

## Competing interests

The authors have declared that they have no competing interests.

## Authors' contributions

DL participated in the design of the study, development of the questionnaire used in the study, recruited participants, collected data, performed data analysis, drafted the manuscript, read and approved the final manuscript. CMF participated in the development of the questionnaire and helped in the analysis of data and read and approved the final manuscript. CC conceived of the study, participated in the design and co-ordination, the development of the questionnaire used in the study, performed data analysis, drafted and read, edited and approved the final manuscript.

## References

[B1] Lemaire F Bion J Blanco J Damas P Druml C Falke K Kesecioglu J Larsson A Mancebo J Matamas D Pesenti A Pimentel J Ranieri M The European Union Directive on Clinical Research: Present status of implementation in EU member states' legislations with regard to the incompetent patients Intensive Care Med 2005 17 476 479 15711974 10.1007/s00134-005-2574-8

[B2] Coppolino M Ackerson L Do surrogate decision makers provide accurate consent for intensive care research? Chest 2001 17 603 612 10.1378/chest.119.2.603 11171743

[B3] Perner A Ibsen M Bonde J Attitudes to drug trials among relatives of unconscious intensive care patients BMC anaesthesiol 2010 17 6 10.1186/1471-2253-10-6 PMC2890661 20504325

[B4] Sugarman J Kass NE Goodman SN Perentesis P Fernandes P Faden RR What patients say about medical research IRB 1998 17 1 7 11657084

[B5] Childs C Vail A Leach P Rainey T Protheroe R King AT Brain temperature and outcome after severe traumatic brain injury Neurocrit Care 2006 17 1 5 10.1385/NCC:5:1:1 16960288

[B6] Kim SY Kim HM McCallum C Tariot PN What do people at risk for Alzheimer disease think about surrogate consent for research? Neurology 2005 17 1395 1401 10.1212/01.wnl.0000183144.61428.73 16275826

[B7] U.S. Department of Health, Education and Welfare Ethical principles and guidelines for the protection of human subjects of research 1979 Washington, DC: US Government Printing Office

[B8] Majesko A Hong SY Weissfeld L White DB Identifying family members who may struggle in the role of surrogate decision maker Crit Care Med 2012 17 2281 2286 10.1097/CCM.0b013e3182533317 22809903 PMC3530841

[B9] Baum A Singer JE Baum CS Stress and the environment J Soc Issues 1981 17 4 35 10.1111/j.1540-4560.1981.tb01056.x

[B10] Chlan L Guttormson J Tracy MF Bremer KL Strategies for overcoming site and recruitment challenges in research studies based in intensive care units Am J Crit Care 2009 17 410 417 10.4037/ajcc2009400 19723861 PMC2760326

[B11] Reynolds WW Nelson RM Risk perception and decision processes underlying informed consent to research participation Soc Sci Med 2007 17 2105 2115 10.1016/j.socscimed.2007.06.021 17689846

[B12] Morris MC Nelson RM Randomized, controlled trials as minimal risk: An ethical analysis Crit Care Med 2007 17 940 944 10.1097/01.CCM.0000257333.95528.B8 17255879

[B13] Yu X Clinical differences in nursing between East and West: Implications for Asian nurses Home Health Care Manage Prac 2006 17 420 423 10.1177/1084822306288438

[B14] Yousuf RM Fauzi ARM How SH Rasool AG Rehana K Awareness, knowledge and attitude towards informed consent among doctors in two different cultures in Asia: A cross-sectional comparative study in Malaysia and Kashmir, India Singap Med J 2007 17 559 565 17538757

[B15] Singapore Department of Statistics Census of Population Education 2010 Singapore: Department of Statistics, Ministry of Trade & Industry

[B16] Ciroldi M Cariou A Adrie C Annane D Castelain V Cohen Y Delahaye A Joly LM Galliot R Garrouste-Orgeas M Papazian L Michel F Barnes NK Schlemmer B Pochard F Azoulay E Ability of family members to predict patient's consent to critical care research Intensive Care Med 2007 17 807 813 10.1007/s00134-007-0582-6 17361388

[B17] Szreter SRS The genesis of the Registrar General's social classification of occupations Br J Sociol 1984 17 523 546

